# Supplementary material for: Parasite Load and Site-Specific Parasite Pressure as Determinants of Immune Indices in Two Sympatric Rodent Species
Source: Animals (Basel). 2019 Nov 22;9(12):1015. doi: 10.3390/ani9121015 (PMC6940963; doi:10.3390/ani9121015)
Supplement: Supplementary file 1 [file animals-09-01015-s001.zip › animals-635731-supplementary/Supplementary Table S1 Site characteristics.pdf]

Supplementary Table S1 Site characteristics. Characteristics and parasite load as determined by *Ixodes ricinus* burden based on data from Hofmeester et al. 2017.

Site	Habitat	Undergrowth vegetation[a]	Coordinates[b]	Mean *I. ricinus* larval / nymphal burden
Buunderkamp	Scots pine forest	*Vaccinium myrtillus*	52°00’56’’N 5°44’50’’E	45.6 / 0.88
Herperduin	Mixed forest	*Molinia caerulea*	51°45’33’’N 5°36’53’’E	2.5 / 0.11
Maashorst	Mixed forest	*Deschampsia flexuosa*	51°42’44’’N 5°35’24’’E	9.6 / 0.14
Stameren	Mixed forest	*Deschampsia flexuosa*	52°03’38’’N 5°21’01’’E	11.4 / 0.46

[a] The given plant species was the most dominant species in the herbaceous layer in the 1 hectare plot.

[b] Coordinates given are the coordinates as measured with a handheld GPS (Garmin eTrex 20) in the middle of the 1 hectare plot.
