# Supplementary figures and images for: Parasite Load and Site-Specific Parasite Pressure as Determinants of Immune Indices in Two Sympatric Rodent Species
Source: Animals (Basel). 2019 Nov 22;9(12):1015. doi: 10.3390/ani9121015 (PMC6940963; doi:10.3390/ani9121015)

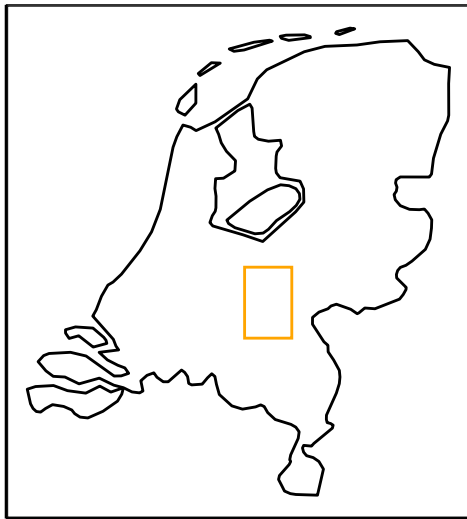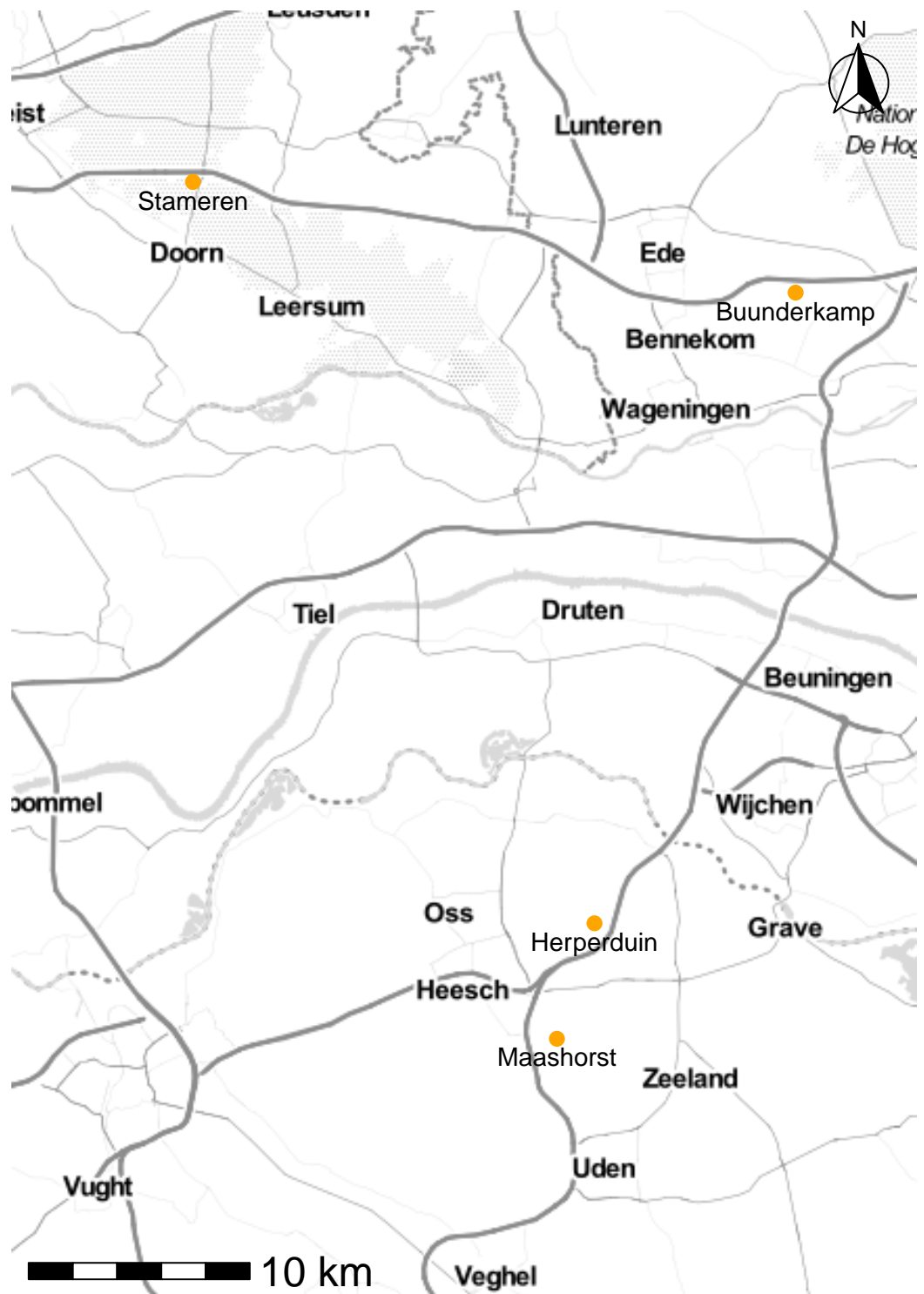

Supplement: Supplementary file 1 [file animals-09-01015-s001.zip › animals-635731-supplementary/Supplementary Figure S1 Map with locations of study sites.pdf]
